# Zero‐Dimensional MXene‐Based Optical Devices for Ultrafast and Ultranarrow Photonics Applications

**DOI:** 10.1002/advs.202002209

**Published:** 2020-09-27

**Authors:** Ning Xu, Hongbo Li, Yiyu Gan, Hualong Chen, Wenjia Li, Feng Zhang, Xiantao Jiang, Yihuan Shi, Jiefeng Liu, Qiao Wen, Han Zhang

**Affiliations:** ^1^ Key Laboratory of Optoelectronic Devices and Systems of Ministry of Education and Guangdong Province College of Physics and Optoelectronic Engineering Shenzhen University Shenzhen 518060 China; ^2^ Shenzhen Engineering Laboratory of Phosphorene and Optoelectronics Collaborative Innovation Center for Optoelectronic Science and Technology Shenzhen University Shenzhen 518060 China

**Keywords:** fiber lasers, MXenes, nonlinear optical materials, quantum dots, ultrafast photonics

## Abstract

In recent years, MXene has become a hotspot because of its good conductivity, strong broadband absorption, and tunable band gap. In this contribution, 0D MXene Ti_3_C_2_T_x_ quantum dots are synthesized by a liquid exfoliation method and a wideband nonlinear optical response from 800 to 1550 nm is studied, which have a larger nonlinear absorption coefficient *β* of –(11.24 ± 0.14) × 10^–2^ cm GW^–1^. The carrier dynamic processes of 0D MXene are explored with ultrahigh time resolution nondegenerate transient absorption (TA) spectroscopy, which indicates that the TA signal reaches its maximum in 1.28 ps. Furthermore, 0D MXene is used to generate ultrashort pulses in erbium or ytterbium‐doped fiber laser cavity. High signal‐to‐noise (72 dB) femtosecond lasers with pulse durations as short as 170 fs with spectrum bandwidth of 14.8 nm are obtained. Finally, an ultranarrow fiber laser based on 0D MXene is also investigated and has a full width at half maximum of only 5 kHz, and the power fluctuation is less than 0.75% of the average power. The experimental works prove that 0D MXene is an excellent SA and has a promising application in ultrafast and ultranarrow photonics.

## Graphical abstract

0D MXene quantum dots are synthesized by a liquid exfoliation method and are used as saturable absorbers to generate ultrashort pulses or ultranarrow linewidth in ultrafast and single‐frequency fiber laser cavity, which have high signal‐to‐noise (72 dB) femtosecond lasers as short as 170 fs and a full width at half maximum of only 5 kHz.

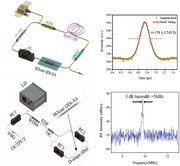

## 1 Introduction

The discovery of graphene opens the door to 2D materials,^[^ ^1^ ^]^ and a large number of 2D materials have been discovered to date, including metals,^[^ ^2^, ^3^ ^]^ semiconductors,^[^ ^4^, ^5^ ^]^ and insulators.^[^ ^6^, ^7^ ^]^ 2D materials have attracted more and more attention because of their unique optical and electrical properties, and the strong interaction between light and materials. Nonlinear optical materials are widely used as saturable absorbers (SAs) to generate passive mode‐locking pulses in lasers.^[^ ^8^, ^9^ ^]^ The generation of ultrafast pulses is widely used in micro‐/nano‐finishing,^[^ ^10^ ^]^ laser detection,^[^ ^11^ ^]^ and laser medical treatment.^[^ ^12^ ^]^ To date, the SA, which is a key device to generate ultrafast pulses, has been occupied by a semiconductor‐saturable absorber mirror (SESAM). SESAM has the advantages of stable operation and low insertion loss, but the adjustable range of SESAM is narrow (generally only dozens of nanometers), which is still hard to support very short femtosecond pulses generation, and the preparation process is very complex.^[^ ^13^, ^14^ ^]^ 2D materials can also be used as SAs, such as graphene, transition metal dichalcogenides (TMDCs), and black phosphorus (BP). However, graphene's low absorption coefficient, low damage threshold, and non‐tunable band gap limit the development of graphene as an SA.^[^ ^15^ ^]^ Single‐walled carbon nanotubes (SWCNTs) are an alternative scheme with low cost and simple structure. The wavelength range of the laser depends on the SWCNT radius. However, SWCNT will not produce resonance when it works at a certain wavelength, so the performance of the device will be damaged due to the great doping loss.^[^ ^16^ ^]^ BP has a tunable band gap and high carrier mobility. In addition, BP is a direct‐band semiconductor,^[^ ^17^, ^18^ ^]^ and it can also convert electronic signals into light. However, BP is very unstable and oxidizes quickly in the air, which seriously affects the characteristics of the material.

Recently, 2D transition metal carbides and nitrides (MXenes) have attracted widespread attention.^[^ ^19^ ^]^ The surface of MXenes is rich in functional groups such as F, O, or OH. Flexible surface group regulation and layered structure give the material good surface hydrophilicity. In addition, MXenes have a tunable band gap, wideband absorption, and high carrier mobility, making it promising in photoelectronics, catalysis, and energy storage showing great potential value. Based on the previous work of the research group,^[^ ^20^, ^21^ ^]^ we have further prepared 0D MXene Ti_3_C_2_T*_x_* quantum dots (QDs) using liquid‐phase stripping technology. From previous research work, graphene QDs exhibit strong quantum confinement and edge effects leading to new properties compared with its 2D nanosheets.^[^ ^22^ ^]^ Density functional theory has confirmed that the value of the electronic gap and the absorption gap might be inversely proportional to the size of BP QDs.^[^ ^23^ ^]^ So far, QDs have been used in photoluminescence, photocatalysis, and energy conversion because of their unique quantum confinement effect.^[^ ^24^, ^25^ ^]^ Up to now, a variety of QD materials have been proven to be SAs to generate ultrafast and ultranarrow pulses, such as carbon QDs,^[^ ^26^ ^]^ BP QDs,^[^ ^27^ ^]^ and PbO QDs.^[^ ^28^ ^]^ Although there have been many reports of MXene nanosheets being used as SAs to generate ultrafast pulses, no reports of MXene QDs in the direction of ultrafast pulses have been found. In addition, 2D or 0D MXene has not been exploited in ultranarrow lasers, which occur widely in the areas of fluorescent probe,^[^ ^29^, ^30^ ^]^ white light‐emitting diodes,^[^ ^31^ ^]^ supercapacitors,^[^ ^32^ ^]^ etc.

In this work, we synthesized MXene QDs using liquid‐phase exfoliation technology, and then characterized the QDs, including transmission electron microscopy (TEM), atomic force microscopy (AFM), photoluminescence excitation (PLE) spectra, UV–vis absorption spectrum, Raman spectroscopy, and transient absorption (TA) spectroscopy. The results of the characterization showed that we successfully synthesized the QDs. We investigated the nonlinear absorption of the QDs by using open aperture (OA) Z‐scan technique, and the results reveal that the MXene QDs have a wideband of absorption and obtain a large nonlinear absorption coefficient, exceeding the MXene nanosheets. We attribute to the enhancement of nonlinear effects caused by QDs confinement effects, which has been reported in the nonlinear enhancement caused by the size dependence of nanomaterials.^[^ ^33^, ^34^ ^]^ Furthermore, we added a solution of MXene QDs to the side‐polished D‐shaped fiber and formed MXene QDs‐SA in an erbium (Er)‐doped or ytterbium (Yb)‐doped laser cavity. Compared with MXene nanosheet, ultrafast pulses were generated relatively easily, which we attribute to the excellent dispersibility of QDs and the larger contact area with side‐polished fiber. Finally, we also utilized MXene QDs to generate an ultranarrow fiber laser having excellent single frequency (SF) properties and high stability, which we also attribute to the outstanding nonlinear optical response of QDs. Our experimental work indicates that MXene QDs are an excellent performance SA and open a new avenue for their application in ultrafast and ultranarrow photonics.

## 2 Morphology and Optical Characterizations of 0D MXene

**Figure 1:**
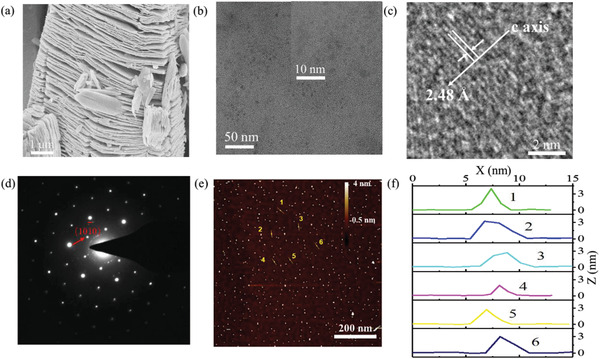
Morphology of MXene Ti_3_C_2_T*_x_* QDs. a) Image of MXene nanosheets. b) TEM image of MXene QDs. c) The interlayer distance is measured to be 2.48 Å. d) SAED image of MXene QDs. e) The AFM image and illustration of MXene QDs are two lines randomly selected. f) The height profiles along the six lines in (e).

In order to easily obtain the MXene QDs, we used a powder MAX precursor with a small size (200 mesh, 11 Technology Co., Ltd., China). The hydrofluoric acid (HF) has a relatively obvious etching effect on MAX phase. We first obtained MXene nanosheets by liquid‐phase exfoliation technique. After a series of preparation processes, we obtained MXene QDs. The schematic diagram of the experimental preparation process is shown in Figure S1, Supporting Information. The TEM (JEM‐3200FS, Japan) image presented in **Figure** **1**b obviously shows the morphology of MXene QDs, and the high‐resolution transmission electron microscopy (HRTEM) image indicates the interlayer distance is ≈2.48 Å. The corresponding selected area electron diffraction (SAED) pattern with clear hexagonal spots in Figure 1d represents the hexagonal lattice structure. We used AFM (Bruker Multimode 8, USA) to conduct morphological characterization of the MXene QDs and the results are shown in Figure 1e, which indicates that the thickness is relatively small.

**Figure 2:**
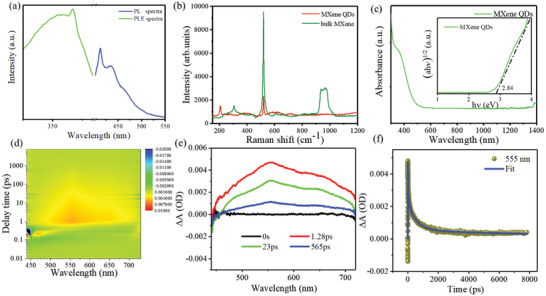
Optical characterization and transient spectra of MXene QDs. a) Excitation and emission spectra of MXene QDs. b) Raman spectra of bulk MXene and MXene QDs. c) UV–vis absorption spectrum of MXene QDs; the inset is the value of the band gap fitted. d) The transient absorption 2D map of MXene QDs. e) Transient absorption spectra of MXene QDs. f) Kinetics at the probe wavelength of 555 nm for MXene QDs.

Since the QDs band gaps are fully open, they fluoresce when exposed to a certain wavelength of light. At present, there are two main reasons for the luminescence mechanism: size effects^[^ ^35^ ^]^ and surface defects.^[^ ^36^ ^]^ We used a fluorescence spectrometer to test the fluorescence characteristics of MXene QDs and obtained their PLE spectra (**Figure** **2**a). The horizontal axis is the excitation wavelength, and the vertical axis is the fluorescence intensity. We used light with wavelengths ranging from 320 to 390 nm to excite MXene QDs without changing the fluorescence wavelength. There is a prominent peak on the spectrum near 367 nm, which indicates that the fluorescence intensity of MXene QDs is the largest under the condition of 367 nm light excitation. The emission spectra of MXene QDs are generally broad and dependent on the excitation wavelength. The strongest peak appears around 415 and 430 nm as excited at 367 nm. Raman spectroscopy was also used to characterize the bulk MXene and MXene QDs. There are six prominent peaks on the spectrum from 200 to 1100 cm (Figure 2b), which is consistent with previous research.^[^ ^37^ ^]^ However, the peak around 500 cm is the signal of Si substrate and it was presented in many test results.^[^ ^38^ ^]^ UV–vis absorption spectroscopy was used to investigate the optical property of the MXene Ti_3_C_2_T*_x_* QDs (Figure 2c). By plotting the absorption to the band edge (put it in Figure 2c), the band gap is determined to be 2.84 eV. The band gap of the initial bulk MXene is relatively small, about 0.1 eV. However, with the decrease of the size, the band gap gradually increases to allow radiation electron transition. For MXene QDs, it is reasonable to increase the band gap through quantum effect to achieve luminescence emission.^[^ ^39^ ^]^ The MXene QDs have a broad absorption ranging from 300 to 1400 nm. There is a peak on the UV–vis absorption spectrum near 370 nm. The inset in Figure 2c is the Tauc plot of MXene QDs. The bandgap calculated from the Tauc plot is 2.84 eV. The peak of the PLE spectrum is consistent with that of the UV–vis absorption spectrum.^[^ ^39^ ^]^

We explored the carrier dynamic possesses of MXene QDs with ultrahigh time resolution nondegenerate TA spectroscopy. Two beams of light are involved in the measurement of TA spectra, including pump light and probe light. The measuring key is to detect the variation of material absorbance about the probe light under the pump light's action. The solvent is isopropyl alcohol (IPA) and the length of the cuvette is 10 mm. The pump power used is 0.18 mW, and the wavelength of the pump light is 420 nm (2.95 eV). An absorption change can be clearly observed as a function of delay time on the spectrum. The transient absorption signal rises when the pump light and the probe light overlap.

Here photon with high energy is employed to excite the electrons to the high energy state. The electrons can be instantly exited in ultrafast time of tens to hundreds of femtoseconds. For the influence of IPA and thick cell, the rise time in this measurement is delayed. Figure 2d shows the transient absorption 2D map of the MXene QDs. The *Y*‐axis represents the pump‐probe delay time in terms of picosecond and the *X*‐axis represents the probe light wavelength with a range from 440 to 760 nm, which indicates that MXene QDs have a broad transient absorption.^[^ ^40^ ^]^

The carrier dynamics in MXene QDs is analyzed basing the TA spectra in Figure 2e, in which the broadband photoinduced absorption (PIA) spectra are observed. Herein, this PIA signal is attributed to the excited‐state absorption (ESA). The TA spectra at selective pump‐probe delay time are shown in Figure 2e. The TA signal reached its maximum in 1.28 ps and then started the cooling relaxation. To further probe the excitation and cooling dynamics, the carrier dynamics at 555 nm is chosen for further analysis (Figure 2f). The data can be fitted by an instrument response function (IRF) correlated exponential function

(1) $$\Delta A\left(t\right)=e^{-{\left(\frac{t-t_{0}}{t_{p}}\right)}^{2}}\times \sum A_{i}e^{-\frac{t-t_{0}}{t_{i}}}$$

where *t*_p_ = IRF/(2ln2) and *t*_0_ is the time zero. Here, four time constants and corresponding decay amplitude are fitted to be *A*_1_ = (1.254 ± 0.093) × 10^−3^, *t*_1_ = 2.615 ± 0.440 ps, *A*_2_ = (1.432 ± 0.094) × 10^−3^, *t*_2_ = 43.3 ± 6.7 ps, *A*_3_ = (1.710 ± 0.094) × 10^−3^, *t*_3_ = 547.6 ± 76 ps, *A*_4_ = (0.562 ± 0.093) × 10^−3^, *t*_4_ = 12.9 ± 6.6 ns. In the MXene quantum dots, a non‐thermal distribution is initially established after excitation, and then the carriers equilibrate themselves by scattering interaction, forming a thermal Dirac–Fermi distribution corresponding time constants *t*_1_. Sequentially, the hot electron and hole transit to respective extrema in the conduction band and valance band during fitting *t*_2_ time via carriers‐optical phonon scatting. Then, electron and hole undergo recombination via fitting time *t*_3_. At last, due to the quantum confinement effect, the propagation of acoustic phonon is limited of size in MXene QDs, and the thermal conductivity decreases. Therefore, the phonon experiences a longer relaxation time of 12.9 ± 6.6 ns, and recovers the initial state. To analyze the material size effect on the carrier dynamics, we also tested MXene nanosheets (NSs) under the same condition of the QDs as shown in Figure S2, Supporting Information. It is worth noting that the TA signal carrier decays faster than for the MXene QDs. The decay process is fitted to be *t* = 189.3 ± 14.0 ps by single‐exponential function. Such a carrier lifetime alignment is caused by the quantum‐confinement‐induced electronic band structure modification. When the morphology of semiconductors is altered to low‐dimensions (0D QDs, 1D nanowire, and 2D film) from the bulk region, the continuous energy band is modified to discrete energy levels.^[^ ^41^, ^42^ ^]^ The energy value between these levels increases significantly, while the phonon energy remains unchanged. Notably, carrier–phonon interaction is an indispensable process during the carrier cooling. As a result, more phonons should participate in cooling a hot electron, which is the phonon‐bottleneck effect. The decreased efficiency of the carrier–phonon interaction causes the prolonged cooling process in low‐dimensional materials. This kind of carrier slowing down phenomenon has been investigated in conventional quantum dots and 2D films, which causing stronger interaction between light and materials.

## 3 Nonlinear Optical Responses of 0D MXene

**Figure 3:**
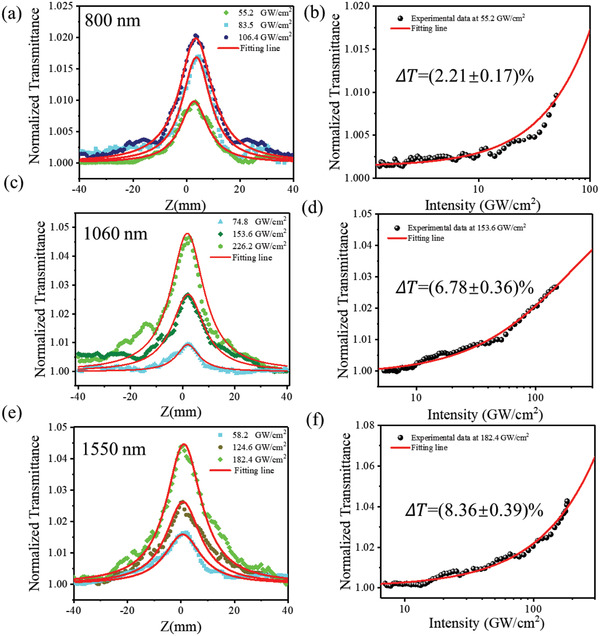
OA Z‐scan characterizations of the MXene QDs at a) 800, c) 1060, and e) 1550 nm. Solid lines are the fitting results with a two‐level energy system model. Normalized transmittance and input peak intensity of the MXene QDs at b) 55.2, d) 153.6, and f) 182.4 GW cm^–2^.

To investigate the broadband responses of MXene QDs and compare the nonlinear optical characteristics of MXene QDs and nanosheets, we utilized an OA Z‐scan technique to measure nonlinear absorption at various wavelengths (**Figure** **3**). A Ti:sapphire oscillator (center wavelength: 800 nm, pulse duration: ≈100 fs, repetition rate: 1 kHz; Spitfire Ace, Spectra‐Physics) and an optical parametric amplifier (TOPAS prime, Spectra‐Physics) system was used. At the slightly higher light intensity, laser beam through the samples will attribute to various nonlinear absorption mechanisms, including ground state bleaching (GSB), ESA, two‐photon absorption (TPA), multi‐photon absorption (MPA), etc.,^[^ ^43^ ^]^ which are typically below picosecond timescale. As shown in Figure 3a–c, we used a two‐lever energy system at 800, 1060, and 1550 nm wavelengths and obtained several better saturable absorption curves with a fitting line. The results demonstrate MXene Ti_3_C_2_T*_x_* QDs are an excellent SA and consistent with previous experimental, such as graphene, BP, and TMDs.^[^ ^44^, ^45^, ^46^, ^47^ ^]^ Materials with such characteristics can be an efficient device for Q‐switching, mode‐locking, and ultranarrow lasers.

The OA Z‐scan technique proposed by Sheik‐Baha et al., can be used to measure not only the nonlinear Kerr effect of materials but also the imaginary part of the third‐order nonlinear polarizability meanwhile.^[^ ^48^ ^]^ So, the absorption coefficient *α* of MXene Ti_3_C_2_T*_x_* QDs can be described as

(2) $$\alpha \left(I\right)=\alpha_{0}+\alpha_{\mathrm{NL}}$$

where *α*_0_ is the linear absorption part, *α*_NL_ = *βI* is the nonlinear absorption part, *β* represents the nonlinear absorption coefficient, and *I* is the incident light intensity. Nonlinear absorption coefficient *β* can be deduced by fitting normalized transmittance *T* versus *z‐*axis using the equation^[^ ^49^ ^]^

(3) $$\begin{array}{ccc} T\left(z\right) & = & \sum_{0}^{\infty }{\left(-\beta I_{0}L_{\mathrm{eff}}\right)}^{m}/{\left(1+z^{2}/z_{0}^{2}\right)}^{m}{\left(m+1\right)}^{\frac{3}{2}}\approx 1\\ & & -\;\beta I_{0}L_{\mathrm{eff}}/2\sqrt{2}\left(1+z^{2}/z_{0}^{2}\right) \end{array}$$

where *I*_0_ is the optical peak intensity at the focus, *L*_eff_ is the effective thickness of the sample, and *z*_0_ is the Rayleigh length of the Gaussian beam. When *β *< 0, absorption coefficient *α* will decrease with the optical intensity increase, that is, the optical intensity transmission of the sample is increased, which is called a saturated absorption.^[^ ^50^ ^]^ As the sample moves from –Z to +Z, the transmittance will appear a peak at the focus. When the sample moves away from the focus, the beam waist radius focusing on the sample is larger, and the power density is smaller, which is mainly linear absorption, and the transmittance curve basically keeps a straight line. When the sample is at the focus, the beam waist radius is the smallest and has a high power density meanwhile, nonlinear absorption and linear absorption of the sample are an order of magnitude, which leads to the largest nonlinear absorption peak. By fitting the measured data with Equation (3), the largest values of *β* were –(3.97 ± 0.03) × 10^−2^, –(6.06 ± 0.39) × 10^−2^, and –(11.24 ± 0.14) × 10^−2^ cm GW^–1^ at 800, 1060, and 1550 nm, respectively. Obviously, it is consistent with the theoretical values of *β* we got. The imaginary part of the third nonlinear optical susceptibility Im*χ* ^3^ depends heavily on *β*. The largest valve of Im*χ* ^3^ is calculated to be –(13.6 ± 0.10) × 10^−13^, –(22.4 ± 0.82) × 10^−13^, and –(38.5 ± 0.48) × 10^−13^ esu at 800, 1060, and 1550 nm, respectively. The value of figure of merit determines how much energy the material can store as a saturated absorber in the resonator. Similarly, we can calculate that the largest values of FOM were –(3.81 ± 0.03) × 10^−13^, –(6.30 ± 0.23) × 10^−13^, and –(10.78 ± 0.13) × 10^−13^ esu cm at 800, 1060, and 1550 nm, respectively.

The MXene Ti_3_C_2_T*_x_* QDs can be fitted using the most common SA model of one‐photon absorption^[^ ^51^ ^]^

(4) $$T=1-\left(\frac{\Delta T}{1+\frac{I}{I_{\mathrm{sat}}}}\right)-T_{\mathrm{ns}}$$

where Δ*T*, *I*_sat_, and *T*_ns_ are the modulation depth, the saturation optical intensity, and the nonsaturable loss, respectively. The largest values of Δ*T* and *I*_sat_ were calculated to be 6.04% ± 0.17%, 9.01% ± 0.21%, and 8.36% ± 0.39%, 106.4, 226.2, and 182.4 GW cm^–2^ at three different wavelengths, whose corresponding single pulse energy was 221, 424, and 380 nJ, respectively. The normalized transmittance and input peak intensity of MXene Ti_3_C_2_T*_x_* QDs are shown in Figure 3d–f.

Before we started the experiments of MXene Ti_3_C_2_T*_x_* QDs, we measured the nonlinear optical properties of solvent (IPA), and the experiment results confirm that the pure solvent not has saturable absorption properties, which is consistent with our previous works.^[^ ^33^, ^52^, ^53^ ^]^ The pure solvent nonlinear optical response is displayed in Figure S3, Supporting Information. Eventually, the MXene QDs linear and nonlinear optical parameters are shown in **Table** **1**, which demonstrates the MXene QDs is an excellent SA. Compared with the previous work (MXene NSs) of our group, our work has a little large value of *β*, which we attribute to the quantum confinement effect of quantum dots to enhance nonlinear effects. Meanwhile, in contrast to other quantum dots of 2D materials, such as graphene, PbS, *β*‐PbO, Fe_2_O_3_, BP, CsPbBr_3_ QDs, etc., the MXene QDs indicates excellent parameters, as shown in **Table** **2**.

**Table 1:** Nonlinear optical parameters of MXene QDs at various wavelengths using OA Z‐scan technique

*λ* [nm]	Δ*T* [%]	*I*_sat_ [GW cm^–2^]	*β* [× 10^–2^ cm GW ^–1^]	*P* [nJ]	*T*_ns_ [%]	Im*χ* ^3^ (× 10^–13^ esu)	FOM (× 10^–13^ esu cm)
800	2.21 ± 0.17	55.2	−(3.97 ± 0.03)	115	25.5	−(13.6 ± 0.10)	−(3.81 ± 0.02)
	5.02 ± 0.10	83.5	−(3.52 ± 0.07)	174	18.2	−(12.0 ± 0.23)	−(3.36 ± 0.06)
	6.04 ± 0.07	106.4	−(2.88 ± 0.06)	221	11.2	−(9.82 ± 0.19)	−(2.75 ± 0.05)
1060	5.68 ± 0.25	74.8	−(6.06 ± 0.39)	156	23.6	−(22.4 ± 0.82)	−(6.30 ± 0.23)
	6.78 ± 0.36	153.6	−(4.27 ± 0.28)	320	17.3	−(15.8 ± 0.96)	−(4.42 ± 0.27)
	9.01 ± 0.21	226.2	−(3.75 ± 0.11)	424	10.4	−(12.7 ± 0.38)	−(3.56 ± 0.11)
1550	3.21 ± 0.20	58.2	−(11.24 ± 0.14)	121	28.6	−(38.5 ± 0.48)	−(10.78 ± 0.13)
	6.07 ± 0.13	124.6	−(6.85 ± 0.07)	259	19.1	−(23.5 ± 0.24)	−(6.58 ± 0.07)
	8.36 ± 0.39	182.4	−(3.03 ± 0.03)	380	12.7	−(10.39 ± 0.14)	−(2.91 ± 0.03)

**Table 2:** Comparison of the nonlinear saturable absorption in various QD materials

Materials	Laser parameters	*β* [cm GW^–1^]	*I*_sat_ [GW cm^–2^]	References
Graphene QDs	532 nm, 21 ps	−1.42 × 10^–2^	117	^[^ ^54^ ^]^
PbS QDs	530 nm, 50 ps	−50	N/A	^[^ ^55^ ^]^
PbS QDs	1060 nm, 50 ps	45.7	N/A	^[^ ^55^ ^]^
*β*‐PbO QDs	1060 nm, 100 fs	−14.95	379.38 ± 46	^[^ ^28^ ^]^
Fe_2_O_3_ QDs	1060 nm, 15 ns	0.82	N/A	^[^ ^56^ ^]^
GaAs QDs	1060 nm, 100 ps	30	N/A	^[^ ^57^ ^]^
BPQDs	800 nm, 100 fs	−(2.5 ± 0.19) × 10^−3^	3.3	^[^ ^58^ ^]^
BPQDs/PMMA	800 nm, 100 fs	−(0.41 ± 0.06) × 10^−3^	21.41 ± 1.87	^[^ ^45^ ^]^
BPQDs/PMMA	400 nm, 100 fs	−(0.29 ± 0.05) × 10^−3^	1.84 ± 0.03	^[^ ^45^ ^]^
CsPbBr_3_ QDs	800 nm, 130 fs	9.7 × 10^−2^	20	^[^ ^59^ ^]^
MXene QDs	1060 nm, 100 fs	−(6.06 ± 0.39) × 10^−2^	74.8	Our work
MXene QDs	1550 nm, 100 fs	−(11.24 ± 0.14) × 10^−2^	58.2	Our work

## 4 Ultrafast Photonics Applications

**Figure 4:**
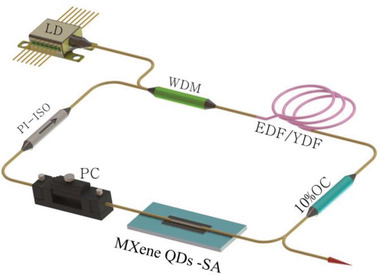
Schematic of passively mode‐locking fiber laser based on MXene (Ti_3_C_2_T*_x_*) QDs‐SA. LD: 980 nm.

The excellent saturation absorption characteristics of MXene QDs indicate that it has a promising application prospect in ultrafast laser photonics. We set up two all‐fiber lasers with Er‐ or Yb‐doped fiber as the laser gain medium. The design of the ring laser cavity is shown in **Figure** **4**. Two types of fiber laser systems with different operational wavelengths were constructed by using Er‐ or Yb‐doped optical fiber (EDF/YDF). In the Er‐doped fiber laser (EDFL), a 4 m Er‐doped fiber (4.45 dB m^–1^ @ 980 nm) and a single‐mode fiber (HI 1060, Corning) are used to construct a ring cavity with a total cavity length of 23.5 m. For the Yb‐doped fiber (250 dB m^–1^ @ 980 nm) and the single‐mode fiber (HI 1060, Corning) to form a ring cavity with the total cavity length of 12.7 m. The EDF/YDF is pumped by a 980 nm laser diode (LD) with a 980/1550 nm (or 980/1060 nm) wavelength division multiplexer (WDM). A polarization‐independent isolator (PI‐ISO) is incorporated to enforce the unidirectional operation of the laser. The cavity polarization state and intracavity birefringence are adjusted by a polarization controller (PC). The MXene QDs solution drops on the side polished of the D‐shaped fiber to form a SA. The MXene (Ti_3_C_2_T*_x_*) QDs‐SA was inserted in between the PC and 10% output coupler (OC). We adjusted the polarization state of the PC, before the SA was inserted into the laser cavity, even if the pump was increased from 0 to 700 mW, and the mode‐locking pulse phenomenon could not be found. Therefore, we eliminate the possibility of self‐starting mode‐locking in the laser cavity for our experiments.

### 4.1 Characterization of the Ultrafast EDFL

**Figure 5:**
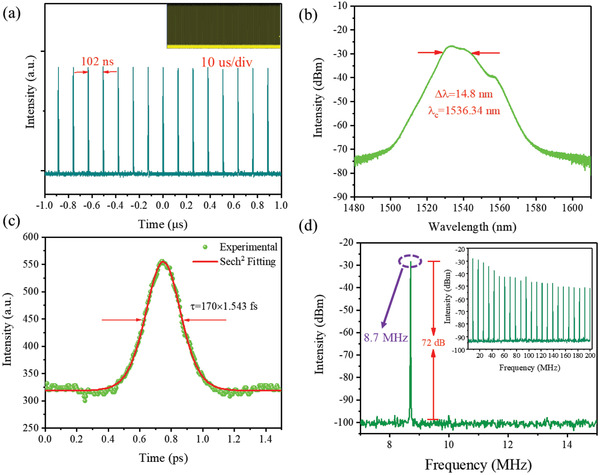
Ultrafast laser output characterizations of EDFL. a) Mode‐locking pulse trains with various time scales. b) Mode‐locking optical spectrum. c) Autocorrelation trace with a sech^2^ fitting. d) The RF optical spectrum at the fundamental frequency; inset: the broadband RF spectrum.

When pump power was increased to 60 mW, the mode‐locking pulse was observed by rotating PC to adjust the polarization state in the intracavity. The characteristics of the EDFL output pulse are shown in **Figure** **5**. The pulse sequence in Figure 5a shows that the interval between each pulse is 102 ns, which matches well with the cavity round‐trip time and confirms that the EDFL is operating at the fundamental mode‐locking state. The insert figure of Figure 5a is the trace measured in a span of 10 µs, which can be certified that the pulse generated in our EDFL was stable in a large span. Figure 5b indicates the optical spectrum of the mode‐locking pulses. The wavelength center is located at 1536.34 nm with 14.8 nm bandwidth at 3 dB. Figure 5c presents the autocorrelation trace of mode‐locking pulse with a full width at half maximum (FWHM) of 260 fs, which means the pulse width is 170 fs if sech^2^ fit is used. Consequently, the corresponding time bandwidth product (TBP) was 0.321, which is very close to the transform‐limited value of the sech^2^ pulse profile (TBP = 0.314), indicating that the soliton pulses were output with little chirping. The radiofrequency (RF) spectrum in Figure 5d reveals the fundamental peak locates at the cavity repetition rate is 8.7 MHz, which corresponds to the total cavity length of 23.5 m. The signal‐to‐noise ratio (SNR) of 72 dB at the resolution bandwidth (RBW) of 1 kHz. The inset of Figure 5d shows that the 0–200 MHz RF spectrum is invariant, revealing that the output laser pulse has a high stability.

**Figure 6:**
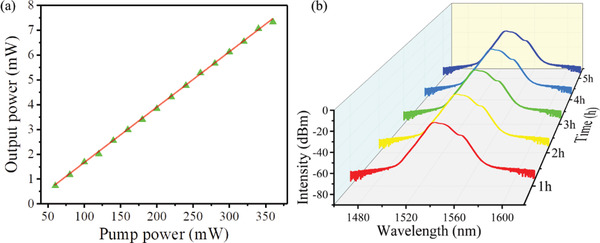
a) The output power varies with pump power. b) Ultrafast laser output optical spectra.

Furthermore, we can still observe the stable mode‐locking pulse output even under a high pump power of 360 mW, which also proves the excellent saturable absorption of MXene QDs. The output power, maximum single pulse energy, and peak power at pump power of 360 mW are 7.33 mW, 0.843 nJ, and 4958 W, respectively. As shown in **Table** **3**, our laser peak power is highest among the ultrafast pulse fiber lasers based on 0D materials. **Figure** **6**a indicates the slope efficiency is 2.20%. Meanwhile, the mode‐locking spectrum was recorded over a period of 5 h. The spectra were very stable with a small change in the central wavelength of only 0.12 nm, as shown in Figure 6b.

**Table 3:** Comparison of output performance of mode‐locking fiber lasers based on various QD materials

Materials	3 dB bandwidth [nm]	Repetition rate [MHz]	Central wavelength [nm]	Pulse duration [ps]	Pump power [mW]	Output power [mW]	Peak power [W]	References
BP QDs‐PMMA	3.4	11.01	1567.6	1.007	70	–	–	^[^ ^45^ ^]^
BP QDs	4.5	15.25	1567.5	1.67	–	–	–	^[^ ^60^ ^]^
PbS QDs	4.78	13.9	1563	0.559	1000	23.5	923	^[^ ^61^ ^]^
CdSe QDs	0.5	14.5	1090	–	468	58.9	–	^[^ ^62^ ^]^
GaTe QDs	–	11.73	1030.7	752	340	9	1.02	^[^ ^25^ ^]^
GaTe QDs	18.1	8.79	1530.9	0.115	345	3.84	3798	^[^ ^25^ ^]^
NbSe_2_ QDs	2.45	7.7	1556	0.756	200	8.3	1426	^[^ ^63^ ^]^
NbSe_2_ QDs	0.155	12.3	1033	380	375	13.8	2.95	^[^ ^63^ ^]^
CsPbBr_3_ QDs	4.5	8.53	1600	14.4	130	5.16	42	^[^ ^64^ ^]^
MXene QDs	7.3	16	1036.9	182	500	13.47	4.63	Our work
MXene QDs	14.8	8.7	1536.3	0.17	360	7.33	4958	Our work

### 4.2 Characterization of the Ultrafast Yb‐Doped Fiber Laser

**Figure 7:**
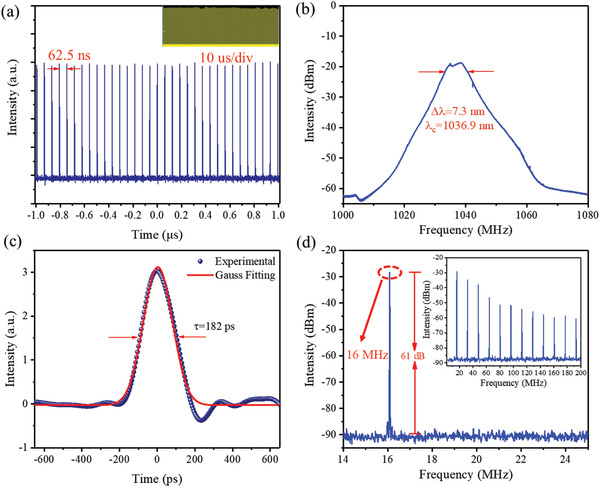
Ultrafast mode‐locking pulse output characterizations of Yb‐doped fiber laser. a) Mode‐locking pulse trains with various time scales. b) Mode‐locking optical spectrum. c) Mode‐locking pulse duration characterized via an oscilloscope. d) The RF optical spectrum at the fundamental frequency; inset: the broadband RF spectrum.

After carefully adjusting the polarization state through the PC, the mode‐locking pulse at 1 µm can also be obtained in the Yb‐doped ring cavity fiber laser based on MXene QDs‐SA. It is worth noting that we have also carried out the laser pre‐experiment of Yb‐doped fiber to prove that there is no mode‐locking phenomenon without the SA in the laser cavity. Stable mode‐locking operation is achieved when the pump power increases to 200 mW. The characteristics of the Yb‐doped fiber laser output pulse are shown in **Figure** **7**. The oscilloscope trace of the mode‐locking pulse train plotted in Figure 7a, with pulses interval of 62.5 ns, which agrees well with the total cavity length of 12.7 m. The insert figure of Figure 7a was the trace measured in a span of 10 µs, which can be attested that the pulse generated in Yb‐doped fiber laser was stable in a large span. Figure 7b shows the optical spectra of the mode‐locking pulses. As shown, the central wavelength is located at 1036.9 nm, and the measured 3 dB bandwidth is 7.3 nm. The single pulse envelope is illustrated in Figure 7c, and the FWHM of the Gaussian function fitting shows the single pulse duration of 182 ps corresponding to a time‐bandwidth product of 371, which indicates that the optical pulse is seriously chirped. The RF spectrum of Yb‐doped mode‐locking fiber laser is shown in Figure 7d. The RF spectrum is located at a fundamental repetition frequency of 16 MHz with an SNR of ≈61 dB. The insert figure of Figure 7d shows that no addition frequency peaks appear on the wideband RF spectrum up to 200 MHz, which indicates the obtained laser pulses with high stability.

**Figure 8:**
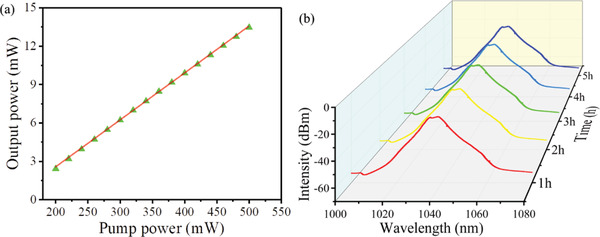
a) The output power of ultrafast Yb‐doped fiber laser varies with pump power. b) Stability of mode‐locking pulse output optical spectra.

Similarly, the Yb‐doped fiber laser output power is a function of the pump power, as given in **Figure** **8**a. When the pump power increased from 200 to 500 mW, the corresponding average output power increases from 2.43 to 13.47 mW, the slope efficiency of ultrafast Yb‐doped fiber laser is around 3.70%. The slope efficiency of fiber laser depends on the efficiency of the gain medium and the loss of a laser cavity. The Stokes limit of a Yb‐doped fiber is higher than that of an Er‐doped fiber,^[^ ^25^ ^]^ which leads to Yb‐doped fiber having higher skew efficiency. The maximum single pulse energy and peak power at the pump power of 500 mW are 0.842 nJ and 4.63 W, respectively. The stability of the Yb‐doped fiber laser was examined by continuously monitoring its output spectra at 1 h intervals over 5 h, and the results are given in Figure 8b. It shows no change in the output spectrum over 5 h. Compared with the mode‐locking output characteristics of other 2D materials QDs (such as BP, PbS, CdSe, GaTe, Nb_2_Se_2_, and CsPbBr_3_), our works show a larger 3 dB bandwidth, higher peak power, and narrower mode‐locking pulse duration (shown in Table 3). It demonstrates that MXene QDs have an excellent performance in a mode‐locking fiber laser, which can contribute to the development of ultrafast optics.

## 5 Ultranarrow Photonics Applications

**Figure 9:**
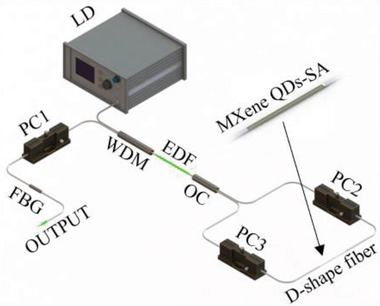
Experimental setup of the SF fiber laser.

In addition, we also applied MXene QDs to SF fiber lasers. The schematic diagram of the proposed ultranarrow linewidth fiber laser is shown in **Figure** **9**. It is constructed in a linear cavity configuration consisting of four parts: a fiber Bragg grating (FBG), a WDM, a segment of EDF, and a fiber loop mirror (LMF). This FBG has a 3 dB bandwidth of 0.036 nm, and its corresponding central reflectivity of 56.95% at a Bragg wavelength of 1549.68 nm. It is not only the output mirror of the proposed SF fiber laser, but also play a significant role in filtering the oscillating light of this linear cavity. The laser can be generated when the 980 nm pump light transmits into the EDF (80 dB m^–1^ @980 nm) via a WDM (980/1550 nm). As it enters the LMF, the laser will be split into two equal‐intensity parts by the 50:50 coupler and transmit in the opposite direction around the loop. A 20 mm D‐shaped fiber is the connection of two output ports of the coupler, and the container of the MXene QDs solution. As shown in Figure 9, to keep laser transmitting in the loop and form the interference of standing wave, we are supposed to adjust the PC2 and PC3 carefully. Attributing to the characters of the SA, MXene QDs solution has less absorption at the central frequency and more absorption at other frequencies. After adding the MXene QDs solution into the D‐shaped fiber, the LMF can be regarded as an ultranarrow bandwidth FBG or a transient grating, and only ultranarrow linewidth light can pass it. Because of the spatial hole‐burning (SHB) effect in EDF, it is necessary for us to adjust PC1 to suppress the effect and eliminate the multimode oscillations. After all of these, a stable SF fiber laser can be achieved successfully.

**Figure 10:**
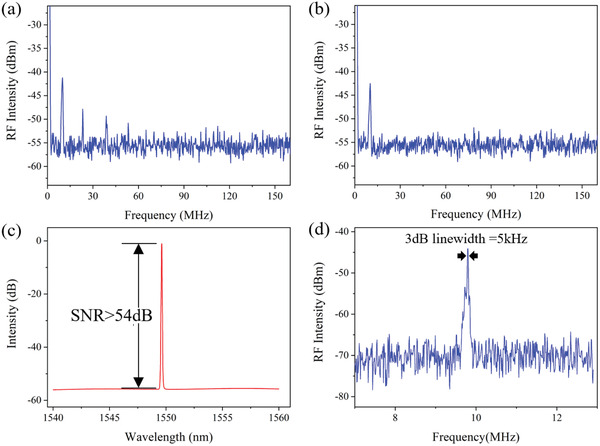
SF output characterizations of a fiber laser. a) Multi‐longitudinal mode oscillation without SA. b) Single longitudinal mode oscillation with SA. c) The SNR of the fiber laser is more than 54 dB. d) The 3 dB linewidth of the SF laser is 5 kHz.

The spectral linewidth of the SF fiber laser is measured by a homemade delay self‐heterodyne system. It consists of a 1 × 2, 50:50 fiber couplers, a 1 × 2, 20:80 fiber coupler (port 80 is connected to the delay line to eliminate the differences of the light intensity between two arms of the system), and a 50 km single‐mode fiber delay line. **Figure** **10**a shows the heterodyne RF spectrum when the MXene QDs solution has not been added into D‐shaped fiber. It is evident that there are several and unstable peaks in the frequency range of 0–160 MHz, when the power of the pump source is 200 mW. It is useless to adjust PC2 and PC3 for achieving the SF output, and the fiber laser still operates at the multimode state. This means that we cannot achieve a SF laser by only using the ultranarrow band filter. We had to add extra SA for a SF output. Then we added SA evenly and sealed it for avoiding the external disturbances. In order to make sure that the light in two arms transmitting in opposite directions can generate interference, we are supposed to adjust the PC2 and PC3 carefully. Figure 10b shows that there is only one peak at 9.73 MHz in the whole scan range, which means that the filter range of transient filter with SA is narrower than the frequency spacing of two adjacent longitudinal modes. As is shown in Figure 10c, the typical emission spectrum of the SF fiber laser with the scanning range of 20 nm is measured by an optical spectrum analyzer (OSA, AQ6370D). Its central wavelength is about 1549.64 nm at the output power of 3.44 mW, as well as its optical SNR is more than 54 dB. The heterodyne RF spectrum is shown in Figure 10d, in which we take 3 dB down from the maximum value and observe a 3 dB bandwidth of 10 kHz. Its corresponding linewidth of the Lorentzian FWHM is only 5 kHz. Compared to the other SF fiber laser, the output 3 dB linewidth is the second narrow of them based on various 2D materials, as shown in **Table** **4**.

**Table 4:** Comparison of output performance of SF fiber lasers based on various 2D materials

Materials	Central wavelength [nm]	3 dB bandwidth [kHz]	SNR [dB]	Power fluctuation	References
Graphene	1553.88	206.25	68.3	–	^[^ ^65^ ^]^
Graphene	1544.66 1545.36	7.27 6.83	50	–	^[^ ^66^ ^]^
Graphene	1064	–	60	–	^[^ ^67^ ^]^
TI:Bi_2_Te_3_	1542.3	10	47.5	–	^[^ ^68^ ^]^
Ni‐MOF	1549.9	3.2	52	<1.3%	^[^ ^69^ ^]^
MoS_2_	1063.88	5.89	60	<2.7%	^[^ ^70^ ^]^
MXene QDs	1549.64	5.0	54	<0.7%	Our work

**Figure 11:**
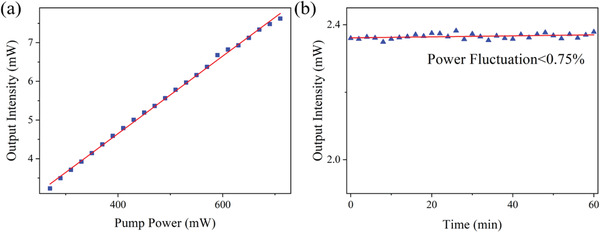
a) The slope efficiency of the SF laser is 1.00%. b) The power fluctuation is less than 0.75%.

From **Figure** **11**a, we can see that the threshold of SF fiber laser is around 262 mW, and when the pump power exceeds the threshold, the laser output power enhances linearly. The maximum output power of the SF laser reaches about 7.6 mW when the pump power is 710 mW. The output power of the SF laser becomes more stable when the fiber laser has worked for a while. As shown in Figure 11b, the power fluctuation less than 0.75% of the average power during 1 h can be easily observed. According to Table 4, compared to the other SF fiber laser, our fiber laser has the most stable output.

## 6 Conclusions

In this contribution, we have synthesized 0D MXene Ti_3_C_2_T*_x_* QDs by liquid‐phase exfoliation technique. The excellent broadband‐saturable absorption properties have been studied from 800 to 1550 nm and show a nonlinear absorption coefficient *β* of –(11.24 ± 0.14) × 10^–2^ cm GW^–1^ and the third nonlinear optical susceptibility Im*χ* ^3^ of –(38.5 ± 0.48) × 10^–13^ esu. Compared with previous works of MXene nanosheet and the other 2D materials, our work has a lager value of *β* and we attribute it to the quantum confinement effects of QDs materials. The carrier dynamic possesses of 0D MXene QDs own longer relaxation time compared with MXene nanosheets, which indicates stronger interaction between light and MXene QDs. In addition, to the best of our knowledge, we for the first time demonstrated MXene QDs as a mode‐locker in our fiber laser cavity with Er‐doped or Yb‐doped fiber laser cavity to generate ultrafast pulses. The EDFL works at a highly stable mode‐locking operation with a duration as short as 170 fs, and its maximum peak power is 4958 W, which is the highest among the ultrafast pulse fiber lasers based on 0D materials. Similarly, the output characteristics of the mode‐locking pulse in the Yb‐doped fiber laser are a duration of 182 ps. Finally, to the best of our knowledge, we also for the first time investigated an ultranarrow fiber laser based on 0D MXene. The ultranarrow laser has a FWHM of only 5 kHz, and its power fluctuation is less than 0.75% of the average power, which is the most stable output of a SF fiber laser based on nanometer materials. Although the research on MXene QDs is in its infancy, we can improve its stability in SA applications through inkjet printing technology, and the size of QDs is also more in line with the requirements of inkjet printing. Our experimental results show that 0D MXene Ti_3_C_2_T*_x_* QDs is an excellent SA and have a promising application for ultrafast and ultranarrow lasers.

## 7 Experimental Section

0D MXene QDs were fabricated by a liquid‐phase exfoliation technique. The schematic diagram of the experimental preparation process is shown in Figure S1, Supporting Information. The purchased MAX (200 mesh, 11 Technology Co., Ltd., China) and HF (Macklin, GR, 40.0%) were fully reacted for 24 h with a magnetic rotor and set a speed of 500 rpm, and the solution of MXene Ti_3_C_2_T*_x_* was obtained. The obtained solution was washed via deionized water until pH > 6, and then dissolved in IPA (Macklin, AR, 99.5%) and probe sonicated (ultrasonic homogenizer, JY92‐IIN, Ningbo, China) for 24 h. Then, the solution was separated with a speed of 5000 and 10 000 rpm by using a high‐speed refrigerated centrifuge (GL‐23M, Xiangyi, China): 5000 rpm for 20 min and 10 000 rpm for 40 min. Taking the supernatant of the high‐speed centrifugation solution and characterizing it, it was confirmed that a good QD material was obtained. The above experimental steps were repeated until 1000 mL of QD solution was collected. Eventually, the collected QD solution was concentrated to 15 mL to obtain a high concentration of QD solution.

## Conflict of Interest

The authors declare no conflict of interest.

## Supporting information

Supporting Information
