# Supplementary material for: Zero‐Dimensional MXene‐Based Optical Devices for Ultrafast and Ultranarrow Photonics Applications
Source: Adv Sci (Weinh). 2020 Sep 27;7(22):2002209. doi: 10.1002/advs.202002209 (PMC7675195; doi:10.1002/advs.202002209)
Supplement: Supplementary file 1 — Supporting Information [file ADVS-7-2002209-s001.pdf]

© 2020 Wiley-VCH GmbH

ADVANCED SCIENCE
Open Access

Supporting Information

for *Adv. Sci.,* DOI: 10.1002/advs.202002209

Zero-dimensional MXene based optical devices for ultrafast and ultra-narrow photonics applications

Ning Xu, Hongbo Li, Yiyu Gan, Hualong Chen, Wenjia Li, Feng Zhang, Xiantao Jiang, Yihuan Shi, Jiefeng Liu, Qiao Wen, and Han Zhang**

Zero-dimensional MXene based optical devices for ultrafast and ultra-narrow photonics applications

Ning Xu, Hongbo Li, Yiyu Gan, Hualong Chen, Wenjia Li, Feng Zhang, Xiantao Jiang, Yihuan Shi, Jiefeng Liu, Qiao Wen, and Han Zhang**

Ning. X, H. Li, Y. Gan, H. Chen, W. Li, F. Zhang, Y. Shi, J. Liu, Assoc. Prof. Q. Wen

Key Laboratory of Optoelectronic Devices and Systems of Ministry of Education and Guangdong Province, College of Physics and Optoelectronic Engineering, Shenzhen University, Shenzhen 518060, China, E-mail: wenqiao@szu.edu.cn

H. Li, H. Chen, F. Zhang, X. Jiang, J. Liu, prof. H. Zhang

Shenzhen Engineering Laboratory of Phosphorene and Optoelectronics, Collaborative Innovation Center for Optoelectronic Science and Technology, Shenzhen University, Shenzhen 518060, China, E-mail:hzhang@szu.edu.cn

Keywords: MXene, quantum dots; nonlinear optical; fiber lasers, ultrafast photonics, ultra-narrow photonics

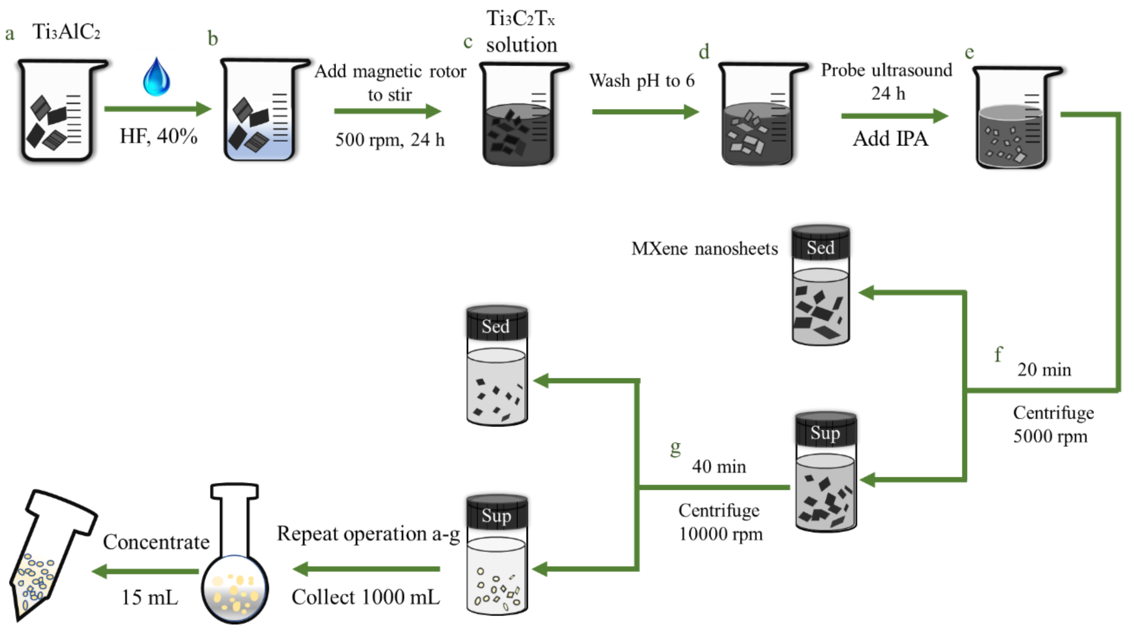

Fig S1. The schematic diagram of the experimental preparation process. Firstly, the bulk MAX $Ti_3AlC_2$was fully reacted for 24 h with HF. Next, the obtained solution was washed Ph > 6 with deionized water, then IPA was added and sonicated for 24 h. The solution after sonication was separated with a speed of 5000 rpm and 10000 rpm by using a high-speed refrigerated

centrifuge,5000 rpm for 20 min and 10000 rpm for 40min. Repeating the experimental steps a-g to collect 1000 mL of MXene QDs solution and concentrate to 15 mL.

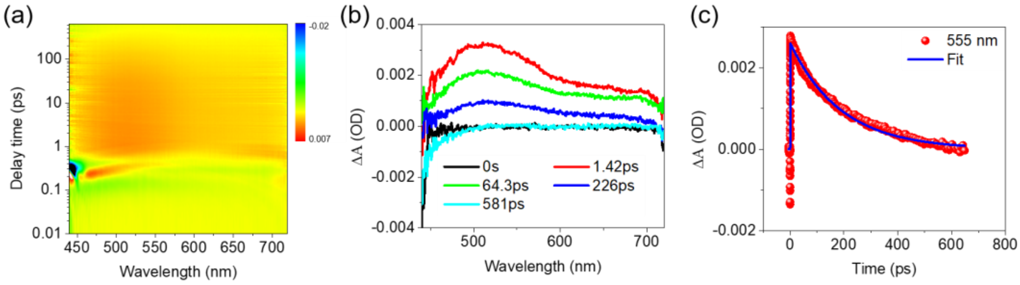

Fig. S2. (a) The transient absorption 2D map of MXene NSs. (b) Transient absorption spectra of MXene NSs. (c)Kinetics at the probe wavelength of 555 nm for MXene NSs.

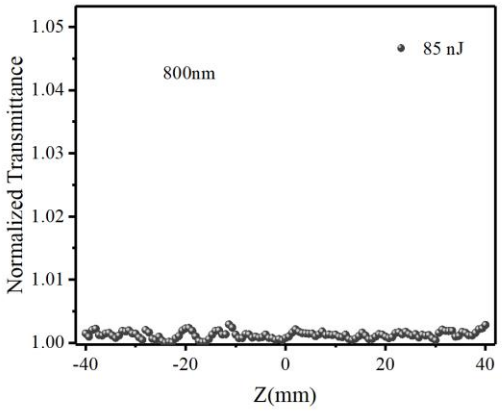

Fig S3. Nonlinear optical response of pure IPA solvent obtained from the OA Z-scan experiment at 800 nm.

Table S1. The concentration and molar absorption rate (absorption coefficient) of QDs vary with the incident wavelength.

λ (nm)	Abs (a.u.)	α_0 (cm^{-1})
426.5	0.174	0.401
431	0.159	0.366
433	0.152	0.350
434.5	0.147	0.338
436	0.142	0.327
330	0.136	0.313

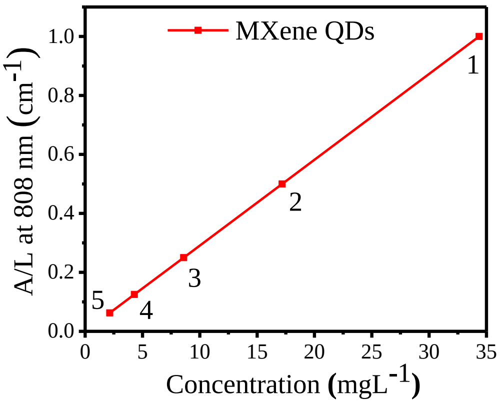

Fig S4. Beer-Lambert law absorbance plot for absorption at 808 nm. A/L=αC, where α is the mass extinction coefficient at 808 nm and its value is 29.1 Lg^{-1} cm^{-1}. Finally, the concentration of QDs calculated is 34.8 mgL^{-1}[1].

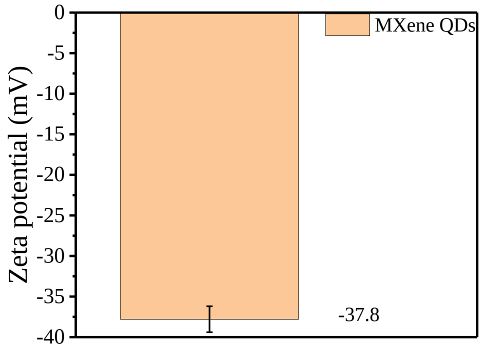

Fig S5. Zeta potential of MXene QDs in IPA. The average value of our three tests is -37.8 mV, and the variance is 1.6 mV. This shows that the dispersion of QDs in IPA is stable[2].

Some equations about the nonlinear optical parameter we listed as follows. The linear absorption part has relations with optical intensity, which can be obtained at low intensity using the equation:

$$\alpha_0 = \frac{\ln(LT)}{LT} \tag{1}$$

where LT is the linear transmittance, and L is the distance of the laser beam through the solution, which is the length of the cuvette (1mm).

The effective thickness of the sample can be calculated by:

$$L_{eff} = 1 - e^{-\alpha_0 L} / \alpha_0 \tag{2}$$

The Rayleigh length of the Gaussian beam can be calculated by:

$$z_0 = \pi w_0{}^2 / \lambda \tag{3}$$

where w_0 is the beam waist, λ is the wavelength of an incident laser beam, our samples (about 220μm) are smaller than the Rayleigh length?

The imaginary part of the third nonlinear optical susceptibility $\mathrm{Im}\chi^3$ is depend heavily on β and the equation is:

$$\mathrm{Im}\chi^3 = \frac{2\varepsilon_0 c^2 n_0^2}{3w}\beta \tag{4}$$

where c is the speed of light in vacuum, ε_0 is the vacuum permittivity, n_0 is linear refractive index ($n_0 \approx 1.73$ for IPA solvent)[3], and w is the angular frequency of the light.

Finally, a figure of merit (FOM) for the third-order optical nonlinearity and linear coefficients can be defined as[4]:

$$\mathrm{FOM} = \frac{Im\chi^3}{\alpha_0} \tag{5}$$

Table S2. Comparison of nonlinear optical parameters in MXene materials.

Materials type	Laser parameters	β(cm/GW)	I_{sat}	Ref
Nanosheets	1064 nm, 20 ps	--	0.26 MW/cm^2	[5]
Nanosheets	1560 nm, 650 fs	--	191 MW/cm^2	[6]
Nanosheets	800 nm, 100 fs	-(5.58±0.19)×10^{-2}	129.08±7.64 GW/cm^2	[7]
Nanosheets	800 nm, 100 fs	11.7×10^{-3}	88.6±5 GW/cm^2	[8]
Nanosheets	--	--	256.9 MW/cm^2	[9]
Nanosheets	800 nm, 100 fs	--	3.7 GW/cm^2	[10]
Nanosheets	800 nm, 100 fs	(8.9±0.02)×10^{-3}	--	[11]
Nanosheets	1557 nm, 400 fs	--	10.68 MW/cm^2	[12]
Nanosheets	1900 nm, 1 ps	--	651.23 MW/cm^2	[12]
Nanosheets	800 nm, 100 fs	--	43 MW/cm^2	[13]
Nanosheets	800 nm, 100 fs	--	34.3 GW/cm^2	[14]
QDs	800 nm, 100 fs	-(11.24±0.14) ×10^{-2}	58.2 GW/cm^2	This work

Table S3. Summary table of passive mode-locking output properties based on MXene saturable absorber.

Gain medium	Structure type	Transfer method	Center wavelength (nm)	SNR (dB)	Pulse duration	Repetition frequency (MHz)	Ref
Yb	D-shaped fiber	Drop	1037.8	75	792 ps	16.5	[7]
			1051.08	57.1	164.4 ps	11.2	[14]
			1065.89	56	480 ps	18.96	[8]
			1036.9	61	182 ps	16	This work
		Inject printed	1060	63	215 ps	16.07	[15]
Er	D-shaped fiber	Drop	1530.85	47	265 fs	8.46	[7]
			1557	70	603 fs	41.46	[16]
			1565.4	62	5.3 ps	8.25	[14]
			1555.01	62	159 fs	7.28	[8]
			1567.3	70.7	946 fs	8.24	[10]
			1536.34	72	170 fs	8.7	This work
		Inject printed	1550	65	114 fs	11.76	[15]
	Tapered fiber	Drop	1566.9	71	1.28 ps	6.032	[9]
			1564.24	55.2	597.8 fs	17.9	[17]
			1550	62.4	104 fs	20.03	[17]
			1556	65	800 fs	6.22	[18]
	Sandwich structure	Clamping	1567.4	59.93	990 ps	0.0463	[19]
			1565	61	1.37 μs	0.131	[6]
	Reflector	Spin coating	2789	33.1	730.4 ns	--	[14]
Tm	Tapered fiber	Drop	1891.82	62	2.18 ps	5.97	[12]

[1] Z. W. Jinnan Xuan, Yuyan Chen, Dujuan Liang , Liang Cheng, Xiaojing Yang , Zhuang Liu , Renzhi Ma , Takayoshi Sasaki , Fengxia Geng, *Angewandte Chemie-International Edition*. **2016**, 55, 14569.

[2] D. Griffiths, W. Bernt, P. Hole, J. Smith, A. Malloy, B. Carr, *Zeta Potential Measurement of Nanoparticles by Nanoparticle Tracking Analysis (NTA)*, **2011**.

[3] E. Sani, A. Dell'Oro, *Opt. Mater.* **2016**, 60, 137.

[4] B. M. Szydłowska, B. Tywoniuk, W. J. Blau, *ACS Photonics*. **2018**, 5, 3608.

[5] J. Wang, S. Liu, Y. Wang, T. Wang, S. Shang, W. Ren, *J. Mater. Chem. C*. **2020**, 8, 1608.

[6] L. Wang, X. Li, C. Wang, W. Luo, T. Feng, Y. Zhang, H. Zhang, *ChemNanoMat*. **2019**, 5, 1233.

[7] Y. Shi, N. Xu, Q. Wen, *J. Lightwave Technol.* **2020**, 38, 1975.

[8] X. Jiang, S. Liu, W. Liang, S. Luo, Z. He, Y. Ge, H. Wang, R. Cao, F. Zhang, Q. Wen, J. Li, Q. Bao, D. Fan, H. Zhang, *Laser Photon. Rev.* **2018**, 12, 1700229.

[9] J. Feng, X. Li, T. Feng, Y. Wang, J. Liu, H. Zhang, *Annalen der Physik*. **2019**, DOI: 10.1002/andp.2019004377.

[10] J. Li, Z. Zhang, L. Du, L. Miao, J. Yi, B. Huang, Y. Zou, C. Zhao, S. Wen, *Photonics Res.* **2019**, 7.

[11] G. Wang, D. Bennett, C. Zhang, C. Ó Coileáin, M. Liang, N. McEvoy, J. J. Wang, J. Wang, K. Wang, V. Nicolosi, W. J. Blau, *Adv. Opt. Mater.* **2020**, 8.

[12] Z. Wang, H. Li, M. Luo, T. Chen, X. Xia, H. Chen, C. Ma, J. Guo, Z. He, Y. Song, J. Liu, X. Jiang, H. Zhang, *ACS Applied Nano Materials*. **2020**, 3, 3513.

[13] Q. Yang, F. Zhang, N. Zhang, H. Zhang, *Opt. Mater. Express*. **2019**, 9, 1795.

[14] J. Yi, L. Du, J. Li, L. Yang, L. Hu, S. Huang, Y. Dong, L. Miao, S. Wen, V. N. Mochalin, C. Zhao, A. M. Rao, *2D Materials*. **2019**, 6.

[15] X. Jiang, W. Li, T. Hai, R. Yue, Z. Chen, C. Lao, Y. Ge, G. Xie, Q. Wen, H. Zhang, *npj 2D Materials and Applications*. **2019**, 3.

[16] G. Sobon, J. Sotor, I. Pasternak, A. Krajewska, W. Strupinski, K. M. Abramski, *Opt. Express*. **2015**, 23, 9339.

[17] Q. Wu, X. Jin, S. Chen, X. Jiang, Y. Hu, Q. Jiang, L. Wu, J. Li, Z. Zheng, M. Zhang, H. Zhang, *Opt. Express*. **2019**, 27, 10159.

[18] K. Jeong Je, K. Hyerim, K. Chong Min, L. Jae Ha, L. Sang Bae, L. Kwanil, *2018 23rd Opto-Electronics and Communications Conference (OECC)*. **2018**, DOI: 10.1109/oecc.2018.87301332 pp.

[19] H. Ahmad, H. S. M. Albaqawi, N. Yusoff, W. Y. Chong, M. Yasin, *IEEE J. Quantum Electron.* **2020**, 56, 1.
